# Intra-Population Competition during Adaptation to Increased Temperature in an RNA Bacteriophage

**DOI:** 10.3390/ijms22136815

**Published:** 2021-06-24

**Authors:** María Arribas, Ester Lázaro

**Affiliations:** Department of Molecular Evolution, Centro de Astrobiología (CSIC), Instituto Nacional de Técnica Aeroespacial (INTA), Carretera de Ajalvir, Km 4, Torrejón de Ardoz, 28850 Madrid, Spain; arribashm@cab.inta-csic.es

**Keywords:** adaptation, RNA viruses, bacteriophages, Qβ, molecular evolution, clonal interference, population bottlenecks

## Abstract

Evolution of RNA bacteriophages of the family *Leviviridae* is governed by the high error rates of their RNA-dependent RNA polymerases. This fact, together with their large population sizes, leads to the generation of highly heterogeneous populations that adapt rapidly to most changes in the environment. Throughout adaptation, the different mutants that make up a viral population compete with each other in a non-trivial process in which their selective values change over time due to the generation of new mutations. In this work we have characterised the intra-population dynamics of a well-studied levivirus, Qβ, when it is propagated at a higher-than-optimal temperature. Our results show that adapting populations experienced rapid changes that involved the ascent of particular genotypes and the loss of some beneficial mutations of early generation. Artificially reconstructed populations, containing a fraction of the diversity present in actual populations, fixed mutations more rapidly, illustrating how population bottlenecks may guide the adaptive pathways. The conclusion is that, when the availability of beneficial mutations under a particular selective condition is elevated, the final outcome of adaptation depends more on the occasional occurrence of population bottlenecks and how mutations combine in genomes than on the selective value of particular mutations.

## 1. Introduction

Whereas the most relevant feature of DNA phage evolution is genetic mosaicism resulting from recombination between non-identical ancestors [1], RNA phage evolution of the family *Leviviridae* is governed by the high error rate of their RNA-dependent RNA polymerases (RdRp), in the order of 10^−4^ to 10^−6^ errors per nucleotide copied [2,3,4]. As it happens in other RNA viruses, this fact, together with their short generation times and usually large population sizes, gives rise to highly heterogeneous populations, referred to as quasispecies [5,6,7], which are composed of a dynamic mutant spectrum that is responsible for a large part of their properties. The most represented nucleotides at each genomic position allow the determination of a consensus sequence, which can be quite useful to follow the mutations that fix throughout an adaptive process, but which does not inform about the underlying changes and reorganizations within the mutant spectrum. The same consensus sequence can be obtained for different mutant spectra, showing its limitations to define RNA virus populations.

Replication at high error rates precludes the total dominance of the best fit genotype, since the generation of new mutations leads to further diversification of the population, even in the absence of evident selective pressures. The presence of a wide variety of mutants, containing or with the ability to generate in a short time a diversity of beneficial mutations, has been usually assumed to be the basis of the great adaptability of RNA viruses. However, this coexistence of mutants may also have unexpected consequences, as we will detail below.

When error rates and population sizes are low, populations adapt through the generation of beneficial mutations that, in the case that they are not lost by genetic drift, subsequently increase their frequencies until they become fixed. However, in the case of RNA viruses, the higher availability of beneficial mutations implies that, before the first mutation is fixed, new ones can emerge in the same or in other genomes, giving rise to different clonal lineages that compete among them in a process known as clonal interference [8,9,10,11,12,13,14,15,16]. In addition, the value and even the sign of mutations can be modified through epistatic interactions [17,18], the environment, and the composition of the population in which they arise. As a consequence, the process of competition within the mutant spectrum is quite complex and involves frequent long-term polymorphisms that reflect the subsets of genomes in which the population is structured.

To understand how interactions among mutants and mutations influence adaptation, it would be desirable to know the genomic sequences and the associated fitness values for a large number of individual viruses. Currently, next generation sequencing protocols provide information about a large number of genomes from a mutant spectrum [19,20,21,22,23], although the short lengths of the reads obtained by most platforms makes the reconstruction of complete sequences difficult [24,25]. Another disadvantage of this approach is the incapacity to distinguish mutations from defective and viable genomes. A more classical approach consists of the isolation and sequencing of biological clones (lytic plaques generated from single viruses) [11,26,27]. Although the number of viruses that can be analysed by this procedure is limited, it can give relevant information on how mutations combine in the most represented viable genomes and the fitness values they provide, which can be very useful in understanding how the process of within-population competition proceeds throughout adaptation.

In this work we have studied the evolutionary dynamics of bacteriophage Qβ, a member of the *Leviviridae* family whose host is the bacteria *Escherichia coli* [28], when it adapts to a typical environmental change, here being the increase in the replication temperature (in this case from the optimal value of 37 °C to 43 °C). Qβ has a single-stranded, positive-sense RNA genome of 4217 nucleotides that encodes four proteins: A2 (from nucleotide 61 to 1320) that mediates phage binding to the bacterial pili, penetration of the viral genome, and cell lysis; coat (from nucleotide 1344 to 1742) that is the major capsid protein; A1 (from nucleotide 1344 to 2330), a readthrough protein that incorporates in the capsid in a low amount; and the replicase (nucleotides 2352-4118) that copies the RNA genome. We chose temperature as the experimental variable for our studies because it is a relevant selective pressure that influences most biological processes, including the speed of enzymatic reactions and the folding of RNAs and proteins [29,30,31]. Thus, it would be expected that adaptation to changes in temperature takes place through diverse mutations placed in different genes, which makes it a good condition for the study of internal competition in RNA virus populations.

There are a large number of works focused on virus adaptation to changes in temperature [32,33,34,35,36,37,38,39]. In the particular case of Qβ, studies carried out in our group showed that: (i) adaptation to 43 °C entails a fitness cost on replication at 30 °C but not at 37 °C [40], (ii) there is an influence of the standing genetic diversity and the pattern of temperature increase on the speed of adaptation [41,42], and (iii) a mutant with increased resistance to heat shocks in the extracellular medium can also adapt to replicate at 43 °C [43]. Studies carried out by other groups also showed that a set of five silent mutations was necessary for adaptation of a particular Qβ variant to 43.6 °C in a thermally adapted *E. coli* strain [44,45]. During the process, the virus increased the adsorption rate, shortened the latent period and increased the burst size [46]. The high number of coincident mutations identified in all these studies is remarkable, and suggests that most of them are specific of high temperature.

The particular objectives of this work were: (i) to track the changes in the consensus sequences of the evolving populations over time, (ii) to isolate individual phages to determine how mutations combine in their genomes at different points of adaptation, (iii) to establish genotype-fitness relationships that help understand the observed mutational dynamics, (iv) to study the competitive advantages of particular genomes, and (v) to study the effect of reducing the intra-population diversity on adaptation.

Our results showed that adapting populations contained a diverse set of mutants in which multiple mutations combined differently. Mutant spectra were highly dynamic, showing the rapid ascent of some genotypes and the loss of particular mutations with beneficial effects at 43 °C. Estimation of the growth rate of a set of individual viruses isolated at an intermediate point of adaption showed that, although they all had higher values than the ancestral virus, there were no large differences among them that could explain the behaviour observed. In contrast to this, reductions of the intra-population diversity showed the advantages of particular combinations of mutations over others. Overall, this work is a clear example of how competitive interactions among mutants within the mutant spectrum modulates the outcome of adaptation in RNA viruses.

## 2. Results

### 2.1. Adaptation of Bacteriophage Qβ to 43 °C

**Figure 1:**
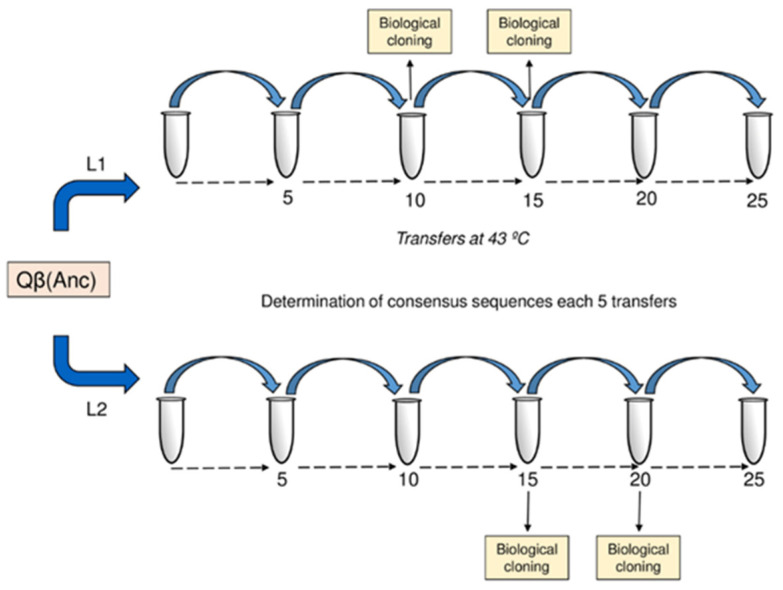
Scheme of the evolution experiment. The figure shows the transfer series experienced by the virus Qβ(Anc) to give rise to the evolutionary lineages L1 and L2, as well as the determinations carried out at different points. The virus was transferred for 25 serial transfers (see the section “Evolution Experiment” in Materials and Methods (Section 4)). Consensus sequences were determined each 5 transfers. At the points shown in the figure, the virus populations were plated to isolate biological clones, and whose sequences were also determined.

The clonal virus Qβ(Anc), obtained from the expression of pBRT7Qβ (see Materials and Methods (Section 4)), was used to found two evolutionary lineages (L1 and L2) that were propagated in parallel at 43 °C for 25 serial transfers (Figure 1). At transfer 25, both populations significantly increased their growth rates at 43 °C, which went from 4.68 ± 0.03 doublings per two hours in the virus Qβ(Anc) to 12.00 ± 0.13 and 12.28 ± 0.16 in L1 and L2, respectively (*p* < 0.001 for the comparison of any of the evolved populations with the virus Qβ(Anc); Student’ *t* test). As expected from previous works [40,44], adaptation to 43 °C did not significantly modify the growth rate at 37 °C, which had values of 14.6 ± 1.6 (Qβ(Anc)), 14.9 ± 0.1 (L1), and 15.3 ± 0.4 (L2) (*p* > 0.05 for the comparison of any of the evolved populations with the virus Qβ(Anc); Student’ *t* test).

A previous experiment, in which a different biological clone, obtained similarly to Qβ(Anc), was propagated at 37 °C for 25 replicated transfers, showed that under this condition the virus did not experience fitness gain at 43 °C [42].

**Figure 2:**
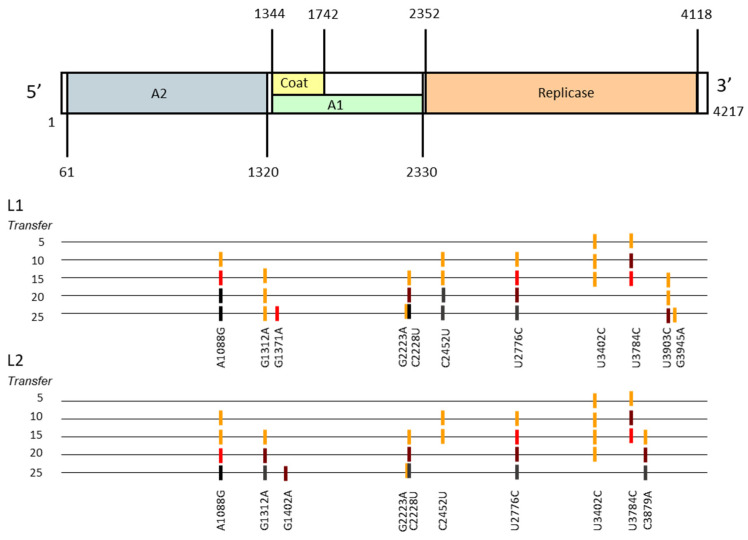
Location in the Qβ genome of the mutations present in the consensus sequences of lineages L1 and L2, propagated at 43 °C for different number of transfers. The upper part of the figure shows a scheme of the Qβ genome with the position of its four genes. The following colour notation was used to indicate the relative abundance of the mutated (M) and wild-type nucleotide (WT): orange (M < WT), red (M = WT), brown (M > WT), and black (the mutation is fixed) (see Materials and Methods (Section 4) for a more complete description).

To identify the genetic changes that occurred through adaptation, we determined the consensus sequences of the populations obtained at different points (transfers 5, 10, 15, 20, and 25), and compared them with the sequence of the virus Qβ(Anc). In the case of polymorphic mutations, the visual inspection of the chromatograms allowed us to estimate qualitatively the relative abundance of the mutated and the wild-type nucleotides (see Materials and Methods (Section 4)). The results (Figure 2) showed that adaptation started similarly in both lineages, with the generation of the polymorphisms U3402C and U3784C (both in the replicase gene), which were already detectable at transfer 5. At transfer 10, mutation U3784C notably increased its representation, at the same time that three additional low intensity polymorphisms were generated in both lineages.

It is remarkable that the first detected mutations disappeared at later transfers; something that was more striking in the case of U3784C, since it was highly represented at transfer 10. Also, substitution C2452U, which was fixed at transfer 20 in L1, was lost in L2. At transfer 25 there were a total of 11 mutations (fixed or polymorphic), of which five were present in both lineages. All of them but U1402A (L2) had been previously identified in other studies in which Qβ was also adapted to high temperature [40,41,42,43,44,45,46]. Mutations distributed in the four genes of Qβ and, with the only exception of C2228U, were non-synonymous.

To discard the possibility that the observed coincidence of mutations between both evolutionary lineages could be due to cross-contamination, we performed a new experiment in which a clonal virus, obtained from expression of pBRT7Qβ, was propagated for 15 transfers at 43 °C in duplicate. Two negative controls, in which bacteria were incubated with culture medium in the absence of virus, were transferred in parallel. The absence of lytic plaques after plating the negative controls indicated that the procedures we used to avoid cross-contamination among samples were working properly (see Table S1 for more detailed information).

The two Qβ lineages propagated at 37 °C only showed two polymorphisms in the consensus sequence (A2187C and C3065U) that did not coincide with any of the mutations that appeared at 43 °C [42].

### 2.2. Selective Advantages of Mutations Selected at High Temperature

To investigate the reasons why, during Qβ adaptation to 43 °C, some mutations in the replicase gene were selected against whereas others were favoured, we generated single mutants through site-directed mutagenesis, and compared the performance of the virus Qβ_U2776C_ (with a substitution that fixed in both lineages) with that of Qβ_U3784C_ and Qβ_U3402C_ (carrying substitutions that were lost at late transfers).

A parallel determination of the growth rates of the three single mutants showed that they had similar values at 43 °C (Qβ_U2776C_: 6.1 ± 0.4, Qβ_U3402C_: 5.9 ± 0.2, and Qβ_U3784C_: 6.3 ± 0.2). In good agreement with previous results obtained for viruses Qβ_U2776C_ and Qβ_U3402C_ [47], the values were significantly higher than that obtained for the virus Qβ(Anc) (3.9 ± 0.8) included in the same assay (*p* < 0.05 for the comparison of any of the single mutants with the virus Qβ(Anc); Student’ *t* test). None of the site-directed mutants showed significant differences to the growth rate of the virus Qβ(Anc) at 37 °C (Qβ(Anc): 15.8 ± 0.4, Qβ_U2776C_: 15.6 ± 0.3, Qβ_U3402C_: 16.0 ± 0.1, and Qβ_U3784C_: 15.4 ± 0.8).

To test whether, as indicated by their growth rates, viruses Qβ_U2776C_, Qβ_U3402C_, and Qβ_U3784C_ also had higher competitive fitness than the wild-type virus, we carried out competition experiments in which equal amounts of each mutant and the virus Qβ(Anc) were propagated together for five transfers at 43 °C. The determination of the consensus sequences of the resulting populations allowed us to know whether one of the viruses became dominant, which would be a clear indicator of its evolutionary advantage. In all cases the single mutant prevailed in the competition, suggesting that the three mutations were beneficial under the conditions used in the experiment: clonal viruses, temperature of 43 °C and absence of additional mutations to those assayed.

To analyse the relative advantages of the mutations, we carried out new competition experiments in which equal amounts of two single mutants were propagated together for five transfers at 43 °C (Qβ_U2776C_ with Qβ_U3784C_, and Qβ_U2776C_ with Qβ_U3402C_). The virus containing U2776C was selected against in the two replicas performed for each competition, showing that the advantage provided by this substitution was lower than those provided by U3402C or U3784C. This result disagrees with the fact that U2776C became fixed in both evolutionary lineages, whereas U3402C and U3784C had a transitory presence.

In previous experiments in which Qβ was propagated at 43 °C in our laboratory, we rarely observed the presence of U3784C in the consensus sequence of the adapted populations. We neither observed the simultaneous fixation of U3402C and U2776C, which suggested that the combinations U2776 + U3402 or U2776 + U3784 in the same genome could confer lower fitness than each mutation separately. To test this possibility, we generated the double mutants (Qβ_U2776C + U3402C_ and Qβ_U2776C + U3784C_) and allowed them to compete for five transfers at 43 °C with equal amounts of each single mutant (Qβ_U2776 + U3402C_ with Qβ_U2776C_ or Qβ_U3402C_ and Qβ_U2776C + U3784C_ with Qβ_U2776C_ or Qβ_U3784C_). In all cases the double mutant was dominant after the competition, indicating that it had higher fitness than the single mutants and, thus, there is no evident reason why these mutations tend not to be associated in the same genome.

### 2.3. Structure of L1 at Intermediate Stages of Adaptation

To analyse how mutations combine in the genomes composing the Qβ adapting populations, we isolated and sequenced a number of biological clones at intermediate stages of the process (L1: 16 clones at transfer 10 and 25 clones at transfer 15; L2: 15 clones at transfer 15 and 16 clones at transfer 20) (Figure 1). Sequences corresponding to viruses from the same population were subjected to a clustering analysis (see Materials and Methods (Section 4)), in order to sort them according to their similarity (Figures S1 and S2).

Most of the clones isolated from L1 at transfer 10 differed in their sequences (see Table 1 for mutations repeated in several genomes and Table S2 for mutations present in only one genome), which gave a value for the density of different haplotypes of 0.94 (see the section “Evolution Experiment” in Materials and Methods (Section 4)). There were 53 mutations that corresponded to 27 nucleotide positions (3.3 mutations per genome on average). No genome contained all the mutations that fixed at transfer 25 (A1088G, C2228U, C2452U, and U2776C; see Figure 2). They were either absent (A1088G) or present at low frequencies (C2452U in two sequences, and C2228U and U2776C only in one; see Table S2). In contrast to this, mutation U3784C, which was selected against at later transfers, was the most represented (81% of the genomes analysed).

The 25 biological clones isolated from L1 at transfer 15 contained 115 mutations located at 23 nucleotide positions (see Table 2 for mutations repeated in several genomes and Table S2 for mutations exclusive of particular genomes). The mean number of mutations per genome was 4.6, higher than at transfer 10, whereas the value for the density of different haplotypes decreased to 0.64. A remarkable result is that the whole set of mutations that fixed at transfer 25 in L1 (Figure 2) was present in 56% of the genomes analysed at transfer 15, five of which also had C3903U (a high intensity polymorphism at transfer 25). These mutations (grey cells in Table 2) were also the most represented at this stage. Looking at the mutations that had a transitory presence in the consensus sequences (U3402C and U3784C; yellow cells in Table 2), the main observation is that they were mostly present in genetic backgrounds in which mutations that fixed at transfer 25 were absent.

The results indicate that from transfer 10 to 15 the mutant spectrum of L1 experienced a great reorganization, which involved a decrease in the intra-population diversity and an increase in the mean number of mutations per genome.

### 2.4. Structure of L2 at Intermediate Stages of Adaptation

The 15 biological clones isolated from L2 at transfer 15 contained a total of 69 mutations located at 21 nucleotide positions (see Table 3 for mutations repeated in several genomes and Table S2 for mutations present in only one genome). Although the mean number of mutations per genome at this transfer was the same as in L1 (4.6), the higher value for the density of different haplotypes (0.87) and the less clear classification of the genomes in well-defined clusters (Figure S3) suggested that, at transfer 15, L2 was more diverse than L1. We did not find any sequence with the complete set of mutations that fixed at transfer 25 (A1088G, G1312A, C2228U, U2776C, and C3879A; grey cells in Table 3).

The 16 biological clones isolated from L2 at transfer number 20 contained a total of 90 mutations located at 28 nucleotide positions, which means an average of 5.6 mutations per genome and a value for the density of different haplotypes of 0.75 (see Table 4 for mutations repeated in several genomes and Table S2 for mutations present in only one genome).

At both transfers analysed, and as it happened in L1, substitutions that disappeared at later stages (C2452U, U3402C and U3784C; yellow cells in Table 3 and Table 4 showed a trend to be present in genetic backgrounds containing few mutations from those that fixed at transfer 25 (grey cells in Table 3 and Table 4). In particular, U3402C and U3784C were never associated to C3879A, and only in a few cases these mutations combined in the same genome with U2776C.

Overall, it seems that the changes experienced in the mutant spectrum of L2 between transfers 15 and 20 resembled those of L1 between transfers 10 and 15. There was an increase in the mean number of mutations per genome and a decrease in the value of the density of different haplotypes. At the same time the genome containing all the mutations that fixed in the consensus sequence at transfer 25 become the most represented at transfer 20 (44%, see Table 4).

A remarkable observation is that many of the mutations that appear in Table 1, Table 2, Table 3 and Table 4, and that did not reach fixation at transfer 25, were also detected in other experiments in which Qβ adapted to 43 °C [40,41,42,43,44]. The same happens with a fraction of the mutations present in single genomes (marked in red in Table S2).

### 2.5. Growth Rate of Mutants Containing Different Combinations of Mutations

**Figure 3:**
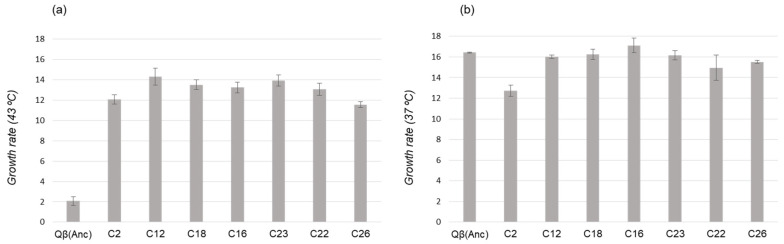
Growth rates of virus clones isolated from L1 at transfer number 15. (**a**) Growth rates determined at 43 °C. (**b**) Growth rates determined at 37 °C. Values were calculated as described in Materials and Methods (Section 4) and expressed as doublings per two hours. The virus Qβ(Anc) was also included in the analysis for comparison. Error bars represent the standard deviation of three parallel replicas.

To investigate whether it is possible to establish relationships between the growth rate values of different mutants and their future representation in the population, we determined this parameter for seven virus clones, chosen among those isolated from L1 at transfer number 15 (Table 2). All clones analysed showed a higher growth rate at 43 °C than the virus Qβ(Anc) (Figure 3a). There were no large differences among clones, although clone C12 (containing all mutations that fix at transfer 25 plus C3903U) had a value slightly higher than the rest, which agrees with the subsequent evolution of L1 (Figure 2).

Clones C23, C18 and C16 had in common the presence of substitutions A1088G, C2228U, C2452U, and U2776C (the same that fixed at transfer 25 in L1), and differed in that U3784C was only present in C23 (Table 2). The similarity in the growth rates at 43 °C of these clones (13.9 ± 0.6, 13.5 ± 0.5, and 13.2 ± 0.5, respectively, *p* > 0.05; Student’ *t* test) suggests that substitution U3784C, which produces a significant increase in the growth rate of the wild-type virus (6.3 ± 0.2 versus 3.9 ± 0.8), does not have a similar effect in this particular genomic context.

Determination of the growth rate values at 37 °C for the same set of virus clones (Figure 3b) showed that, with the only exception of C2, there were no large differences with the virus Qβ(Anc), demonstrating again the absence of trade-offs between adaptation to 43 °C and replication at 37 °C.

### 2.6. Competitive Interactions within the Mutant Spectrum of L1 at Transfer 15

**Figure 4:**
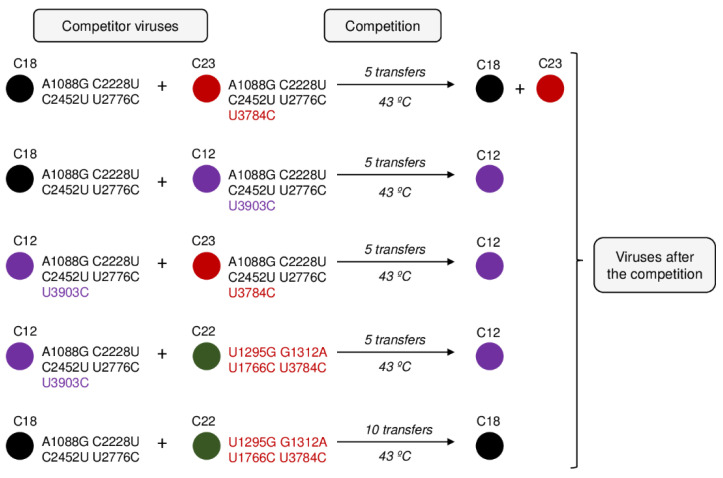
Competitions between pairs of biological clones. All clones were isolated from L1 at transfer 15 (Table 2). Competitions were carried in duplicate as described in Materials and Methods (Section 4). Mutations that were fixed at transfer 25 in L1 are indicated in black, those that remained as polymorphisms at the same transfer are indicated in purple, and those that did not reach fixation or disappeared at later stages are indicated in red.

To better understand the process of internal competition among mutants, we selected some representative clones of L1 at transfer number 15 (Table 2) and performed competition experiments between them (Figure 4). All competitions were replicated.

First, we competed clones C18 and C23, which have in common all mutations that fix at transfer 25 in L1 and differ in the presence of U3784C in the latter. In good agreement with their growth rate values, after five transfers of joint propagation at 43 °C, the two populations obtained were still polymorphic at position U3784C (with similar amounts of the wild-type and the mutated nucleotides), indicating that both viruses had similar competitive fitness. In contrast to this, mutation C3903U did confer an advantage when present in the same mutational context of C18, as was demonstrated by the dominance of C12 after five transfers of joint propagation with C18. As expected from these results, competition between C23 and C12, differing in the presence of U3784C in C23 and C3903U in C12, also led to the dominance of C12.

Finally, we competed clone 22 (containing U3784C accompanied by three mutations that, although are not among those that fix at transfer 25, have been detected in other experiments of Qβ adaptation to high temperature) with either clone C18 or C12. In all cases clone C22 was selected against, although in the case of the competition with C18, 10 transfers were necessary instead of five.

### 2.7. Evolution of Reconstructed Virus Populations

We also analysed the effect of reducing the number of different genomes that coexist in a population on its subsequent adaptation. To this aim, we selected four virus clones from population L1 at transfer number 15 (C3, C16, C22, and C25; see Table 2), and mixed them at equal amounts (10^6^ pfu of each) in duplicate. In this way, we reconstructed two parallel populations that, with a lower number of different genomes than those present in L1, contained most of the mutations that were repeated in the whole set of viruses analysed. Evolution of the reconstructed populations for 10 serial transfers at 43 °C led in both cases to a defined consensus sequence in which substitutions A1088G, C2228U, C2452U, U2776C, and C3903U were imposed. There were no polymorphisms that could be detected through Sanger sequencing. In contrast to this, evolution of L1 also for 10 transfers (from transfer 15 to transfer 25) led to the fixation of the same set of mutations but C3903U and the presence of five additional polymorphisms, one of which was C3903U (Figure 2). The growth rate values of the two reconstructed populations after 10 transfers at 43 °C were 12.8 ± 0.5 and 12.9 ± 0.5, slightly higher than that obtained for L1 at transfer 25 in the same assay (11.7 ± 0.5) (*p* < 0.05 for the comparison of any of the reconstructed populations with L1; Student’ *t* test).

## 3. Discussion

In this work we studied the process of internal competition among mutants that takes place during RNA bacteriophage adaptation, using Qβ as an experimental system and the increase in the replication temperature as the selective pressure.

The two evolutionary lineages analysed showed a clear increase in their growth rate at 43 °C after 25 transfers at this temperature, which is indicative of adaptation. With the only exception of U1402A in L2, all mutations fixed or represented as polymorphisms in the consensus sequences determined each five transfers had been detected in previous studies of adaptation of Qβ to increased temperature [40,41,42,43,44], indicating that they probably have selective value and did not increase their frequency due to hitchhiking. Some of the mutations detected were also identified in Qβ populations propagated at 37 °C (A1088G, G3945A) [41,48,49], which could mean that they may have a more general beneficial effect.

Mutations present in the consensus sequences at the first transfer analysed were the same in both populations: U3402 and U3784. Although we cannot completely discard cross-contamination between lineages, the careful measures we took to avoid this problem make us think that this was not the case. Rather, the coincidence suggests that the pre-standing genetic diversity contained in the ancestral population, which was common for both lineages, was relevant for its subsequent adaptation to 43 °C, favouring the initial selection of the same mutations. Although the ancestral virus corresponded to a lytic plaque generated from a single genome replicating for a few generations, which limits the generation of genetic diversity, the high error rate of Qβ probably permits the coexistence within the plaque of a certain variety of minority genomes. The heterogeneity of subclonal components of lytic plaques has been demonstrated for other RNA viruses [50,51] and agrees with the accelerated fixation of mutations that takes place when they are transmitted through plaque-to-plaque transfers in a process that implies extreme population size reductions at each transfer [52,53,54,55]. In addition, a common observation in our group is that evolutionary lineages that are propagated in parallel from the same ancestral population display a larger number of coincident mutations [40,41,42,43] than when the ancestral population is different. Studies carried out with other viruses also show that evolutionary convergence is a quite common outcome when parallel populations are propagated under the same conditions [56,57,58,59,60]. Determination of the influence of the pre-existent genetic diversity on Qβ adaptation would require analysis of whether the mutations detected at earlier stages were already present as minority variants in the founder population. Such studies are currently being carried out in our group.

The loss of mutations U3402C and U3784C at later transfers was unexpected since both showed higher competitive advantage in the genetic context of the wild-type virus than U2776C, which become fixed. A possible explanation is based on the fact that before any advantageous mutation reaches fixation, others (beneficial or not) may arise in the same or in different genomes. Consequently, an advantageous mutation that is increasing its representation could be displaced by others that are present in more fitting combinations [8,9,10,11,12,13,14,15,16]. In agreement with this idea, the sequences of the virus clones isolated from evolved populations at intermediate stages showed a trend of U3402C and U3784C being present in genetic backgrounds different from the one that fixed at transfer 25 (Table 1, Table 2, Table 3 and Table 4). However, determination of the growth rates of a set of virus clones carrying different combinations of mutations (Figure 3) showed only small differences among them, which do not seem sufficient to explain the advantages of some particular genome over others.

Since the growth rate is not the only trait that may lead to fitness differences [61,62], we analysed the competitive ability of several virus clones (all isolated from L1 at transfer 15), focusing particularly on the effect of the presence of U3784C, whose loss throughout adaptation was much more striking than that of U3402C. The results showed that substitution U3784C provides a higher beneficial effect in the genomic context of the wild-type virus than when it is accompanied by the mutations that fixed at transfer 25 in L1, displaying an example of diminishing return epistasis [63,64]. Other experiments of the same type unveiled the disadvantages of genomes containing U3784C in different mutational contexts (Figure 4). The genome containing all mutations fixed in L1 at transfer 25 plus U3903C seemed to be the fittest among those analysed, which agrees with the increase in the representation of this mutation at later transfers.

A general explanation for the loss of U3784C could be that it induces a shift in the distribution of mutation effects, in such a way that the benefits provided by additional mutations are reduced [65]. The search for the presence of U3784C in previous experiments of Qβ adaptation to high temperature showed that it was only present in 4 lineages out of a total of 37 revised in the literature [40,44,45], showing that, despite its selective advantage when present as single mutation, U3784C is not frequent at late stages of adaptation, when it is more probable that genomes contain a higher number of mutations. A possible explanation could be that this mutation, when accompanied by others, alters the tridimensional structure of the replicase in a way that reduces its functionality and/or stability. We are currently carrying out experiments in which the single mutant Qβ_U3784C_ is being propagated at 43 °C. The presence or not of U3784C in the adapted populations will inform us about how probable the loss of this mutation is as adaptation progresses. The analysis of the mutant spectrum generated from the mutant Qβ_U3784C_ in comparison with that generated from the wild-type virus will provide relevant information concerning the regions of the space of sequences explored in each case.

The ascent of the genotype carrying all the mutations that fixed at transfer 25 took place between transfers 10 and 15 in L1 and between transfers 15 and 20 in L2. In both evolutionary lineages, adaptation was associated with reductions in the genetic diversity that were compatible with the action of natural selection. Given the high error rates of RNA viruses, it would be expected that these reductions in diversity will be transitory. Generation of mutations does not stop and once a set has been fixed, the dominant genotype will continue to diversify. However, it is also likely that large-effect beneficial mutations had already been fixed at earlier stages, which together with the constraints imposed by the new genetic background to the acceptance of additional mutations, may restrict the relevance for adaptation of this late diversity. Broadening of the mutant spectrum in viruses evolving under constant conditions was observed in a recent study carried out with Hepatitis C Virus [23] that showed the coexistence of multiple alternative mutational pathways for fitness gain. However, as the same authors point out, this is not the only possible outcome of RNA virus replication, since mutant spectrum compressions are also frequently observed.

Reduction of the intra-population diversity through the artificial mixing of several representative virus clones isolated from L1 at transfer 15 led to faster fixation of mutations and to slightly higher growth rates than those observed in the original lineage. This finding suggests that populations made up of a high number of different beneficial mutants could adapt faster if occasional population bottlenecks reduced the genetic diversity, and with it the interference and competition among genomes. However, if population bottlenecks occurred too early in adaptation, highly beneficial combinations of mutations could not have the opportunity to be generated, which could trap the population in a low fitness peak with low possibilities for further optimization. All these considerations suggest that in highly diverse populations there is not an optimal population size or an optimal mutation rate for adaptation [66,67,68] but these values change through time, depending on the composition of the mutant spectrum at each moment.

The nature and intensity of the selective pressure is probably another factor determining the importance of internal competition within the mutant spectrum. If the number of beneficial mutations under a particular condition is low, as it happens in the case of mutations providing resistance to some drugs, there are little opportunities for interference. In contrast to this, wide spectrum selective pressures, such as temperature, may induce adaptive responses in most genes, which probably contributes to the increase of the number of different evolutionary pathways that might coexist within a mutant spectrum for a certain time, until a population bottleneck eliminates a fraction of the genetic diversity or a genome with higher selective advantage emerges.

In this manuscript we have pointed to the high error rates and large population sizes of RNA phages as the main sources of interactions within their populations. However, natural environments are much more complex than laboratory contexts and are influenced by a great number of interacting variables. We do not know whether error rates can change depending on factors such as sun radiation, temperature or the metabolic state of the bacterial host, among others. Population size is another element that is easily controlled in the laboratory and that is probably subjected to frequent fluctuations in natural environments. Adverse conditions can cause strong population bottlenecks, which may impair adaptation due to the associated reductions in diversity. Nevertheless, if populations contain many competitor mutants, bottlenecks could be beneficial, by eliminating interference interactions as it is shown in this work. Integration of field work with laboratory studies would be very useful to understand the extent to which studies carried out under controlled conditions could be extrapolated to the real world.

## 4. Materials and Methods

### 4.1. Virus and Bacteria. Standard Procedures for Infection

The plasmid pBRT7Qβ, which contains a cDNA of bacteriophage Qβ cloned in the plasmid pBR322 [69,70], was used to transform *Escherichia coli* DH5-α, a strain that permits virus expression but that cannot be infected, due to lack of the virus receptor. The supernatant of an overnight culture obtained from a transformed colony was used to infect *E. coli*, strain Hfr (Hayes), in semisolid agar. The virus progeny contained in a randomly chosen lytic plaque was used as the ancestor (Qβ(Anc)) of the two evolutionary lineages propagated at 43 °C in this work. The virus Qβ(Anc) showed no mutations relative to the Qβ cDNA cloned in pBR322, and was considered an equivalent to the wild-type virus.

Qβ was propagated in *E. coli* Hfr in NB medium (8 g/L Nutrient Broth from Merck and 5 g/L NaCl). Infections in liquid medium were carried out using fresh log-phase *E. coli* cultures with an OD_550_ between 0.6 and 0.8 that were infected at the multiplicity of infection (moi) indicated in each experiment. After 2 h of incubation either at the optimal (37 °C) or sub-optimal temperature (43 °C) with good aeration (250 rpm), cultures were treated with 1/20 volume of chloroform for 15 min at 37 °C with shaking (300 rpm). Virus supernatants were harvested upon centrifugation at 13,000× *g* and maintained at 4 °C for short-term use (less than 15 days) or at −80 °C for long-term storage. Virus titres were determined by plaque assay and expressed as the number of plaque forming units (pfu) per mL of the phage suspension.

### 4.2. Evolution Experiment

This virus Qβ(Anc) was used to infect two parallel *E. coli* cultures as described above, using an moi of 0.01 pfu/cell in a volume of 10 mL (containing ~10^9^ bacteria). After 2 h of incubation at 43 °C, the virus supernatants were collected, and ~10^7^ pfu of each phage suspension were used to infect fresh *E. coli* cultures, keeping the moi around 0.01 pfu/cell. This procedure was repeated for a total of 25 serial transfers giving rise to two evolutionary lineages, named L1 and L2, whose consensus sequences were determined each 5 transfers (Figure 1). A negative control in which bacteria were incubated in culture medium in the absence of virus was set at each transfer. This control was processed and plated exactly the same as the experimental samples. When lytic plaques appeared, the corresponding transfer was discarded and repeated again.

At two points (transfers 10 and 15 in L1, and transfers 15 and 20 in L2), the virus populations were plated in semisolid agar to isolate biological clones, that were then sequenced. Virus clones were named with the letter C, followed by a number that was assigned consecutively upon their isolation. As a measure of intra-population diversity we used the density of different haplotypes, which was evaluated as the ratio between the number of different sequences identified in each population and the total number of virus clones sequenced. Growth rate values were determined at 37 °C and 43 °C for 7 biological clones isolated from L1 at transfer 15.

### 4.3. Site-Directed Mutagenesis

The plasmid pBRT7Qβ was used to engineer a single-mutant virus, Qβ_U3784C_, containing substitution U3784C. Mutagenesis was carried out using the QuickChange II Site-Directed Mutagenesis Kit (Agilent Technologies, Santa Clara, CA, USA) with the primer 5′CGTCGGATCGGTCCTAACCAATCCTTTCGCG3′ and its complementary that contained the mutation to introduce in the virus genome. The procedures to build and isolate the site-directed mutant were the same as for mutants carrying U2776C (Qβ_U2776C_) and U3402C (Qβ_U3402C_), described previously [47] and also used in this study. For the analysis carried out in this work, a lytic plaque generated in *E. coli* grown at 37 °C by each of the viruses Qβ_U3784C_, Qβ_U2776C_, and Qβ_U3402C_ was picked and sequenced to test the presence of the desired mutation and the absence of any others that might have arisen during the process. The 3 mutants were stable for at least 10 serial transfers carried out either at 37 °C or 43 °C.

The double mutants Qβ_U2776C + U3784C_ and Qβ_U2776C + U3402C_ were prepared following the same protocol described above but using the plasmid pBRT7Qβ containing the mutation U2776C as the substratum for the introduction of mutations U3784C or U3402C.

### 4.4. Isolation of Biological Clones

Biological clones corresponded to lytic plaques obtained in semisolid agar in *E. coli* grown at 37 °C. They were isolated by punching and removing the top and the bottom agar around well-separated lytic plaques as previously described [11,47,48]. The agar containing each plaque was transferred into a tube with 1 mL of phage buffer (1 g/L gelatin, 0.05 M Tris-HCl, pH 7.5, and 0.01 M MgCl_2_) and 50 μL of chloroform, and incubated for 1 h at 25 °C with shaking (900 rpm). After centrifugation at 13,000× *g* for 15 min to clarify the supernatant, the latter was stored over 25 μL of chloroform.

### 4.5. Growth Rate Determinations

Triplicate liquid cultures containing 10^8^ bacteria growing in exponential phase were inoculated with 10^4^ pfu of the virus clone or virus population indicated, in a final volume of 1 mL. After two hours of incubation either at 37 °C or 43 °C, the virus supernatants were collected as described above, and titrated to estimate the virus yield. Growth rate values were expressed as doublings per two hours, and calculated as the log_2_[(*N_f_* − *N_0_*)/*N_0_*], where *N_0_* was the initial input of virus, and *N_f_* was the number of pfu recovered at the end of the assay. When the growth rates of different viruses were compared, their values were always determined in parallel to ensure that the experimental conditions were the same for all.

### 4.6. Competition Experiments

Competitions were carried out by infecting 10^8^ bacteria with equal amounts (between 10^5^–10^6^ pfu) of two virus clones with known sequence in a final volume of 1 mL. After 2 h of incubation at selective temperature (43 °C), the viral supernatant was collected, and a fraction was used to initiate a new infection, keeping the moi around 0.01 pfu/cell. In all cases, competitions were carried out in duplicate. The consensus sequences of the populations obtained after the number of transfers indicated in each case were determined to test whether one of the viruses had outcompeted the other, which was visualized through the inspection of the chromatograms of the nucleotide positions that distinguished the two competitors.

### 4.7. RNA Extraction, cDNA Synthesis, PCR Amplification, and Nucleotide Sequencing

Viral RNA was prepared following standard procedures to determine the consensus sequence either from biological clones or from complex virus populations. RNAs were used for cDNA synthesis with the avian myeloblastosis virus reverse transcriptase (Promega), followed by PCR amplification using Expand high-fidelity DNA polymerase (Roche). In the case of consensus sequences, the following pairs of oligonucleotide primers were used for RT-PCR: P1 forward (5′CTTTAGGGGGTCACCTCACAC3′) with P1 reverse: (5′GGATGGGTCACAAGAACCGT3′) to amplify from nucleotide position 10 to 1595, P2 forward (5′CTCAATCCGCGTGGGGTAAATCC3′) with P2 reverse (5′CAGAAAATCGGCAGTGACGCAACA3′) to amplify from nucleotide position 1407 to 2817, and P3 forward (5′GTGCCATACCGTTTGACT3′) with P3 reverse (5′GATCCCCCTCTCACTCGT3′) to amplify from nucleotide position 2254 to 4195. In the case of biological clones, primers P1 forward and reverse were substituted by P4 forward (5′CGAATCTTCCGACACGCATCC3′) and P4 reverse (5′AAACGGTAACACGCTTCTCCAG3′) that amplified from nucleotide 150 to 1497. PCR products were column purified (Qiagen) and subjected to standard Sanger sequencing using BigDye Chemistry with an automated sequencer (Applied Biosystems; Perkin Elmer; Madrid, Spain ). Sequences were assembled with SeqMan Pro (DNASTAR Lasergene 12 Core Suite) and aligned with Clustal X. Mutations relative to the sequence of the virus Qβ(Anc) were identified using BioEdit. Chromatograms were also visually inspected to test the possible presence of polymorphisms at the nucleotide positions previously identified as relevant for Qβ adaptation at high temperature [40,41,42,43,44]. In the case of polymorphic mutations, we qualitatively determined the relative amount of the mutant and the wild-type nucleotides. When the height of the peaks corresponding to both nucleotides differed by less than 10%, they were considered to be in similar amounts (M = wt). Otherwise, the mutant was considered to be in higher (M > wt) or lower amount (M < wt) than the wild type.

### 4.8. Clustering Analysis

The sequences corresponding to the virus clones isolated at transfers 10 and 15 (L1) and transfers 15 and 20 (L2) were subjected to a clustering analysis that allowed the sorting of them according to their similarity. The analysis was carried out through maximum likelihood methods (PhyML, program Seaview 4, default parameters).

## Figures and Tables

**Table 1:** Mutations present in the genomic sequences of the virus clones isolated from lineage L1 at transfer 10.

Virus Clone ^1^	Mutations Present at Least in Two Virus Clones ^2^									
	1025 U/C	1766 U/C	1817 G/A	1820 G/A	2201 C/U	2261 A/G	2452 C/U	3402 U/C	3784 U/C	3903 C/U
C12		+							+	
C13		+							+	
C3		+								
C9		+					+			
C6					+				+	+
C2	+				+				+	+
C8				+					+	
C1	+			+					+	
C10			+						+	
C16			+				+			
C14			+						+	
C7									+	
C11									+	
C5						+		+	+	
C4						+		+	+	
C15						+		+	+	
Freq ^3^	0.13	0.25	0.19	0.13	0.13	0.19	0.13	0.19	0.81	0.13

^1^ The virus clones analysed were sorted according to the results obtained in a clustering analysis carried out with their genomic sequences (see Materials and Methods (Section 4) and Figure S1). ^2^ The presence of each mutation in the corresponding virus is indicated with a sign +. Blank cells mean the absence of the mutation. Grey cells indicate mutations that were fixed at transfer 25. Yellow cells refer to mutations that had a transitory presence in the consensus sequence throughout the transfer series (see Figure 2). Additional mutations represented in single viruses are indicated in Table S2. ^3^ Frequency reached by the corresponding substitution in the ensemble of clones analysed.

**Table 2:** Mutations present in the genomic sequences of the virus clones isolated from lineage L1 at transfer 15.

Virus Clone ^1^	Mutations Present at Least in Two Virus Clones ^2^											
	1088 A/G	1295 U/G	1312 G/A	1760 C/U	1766 U/C	2223 G/A	2228 C/U	2452 C/U	2776 U/C	3402 U/C	3784 U/C	3903 C/U
C23	+						+	+	+		+	
C21	+						+	+	+			
C9	+						+	+	+			
C5	+						+	+	+			
C13	+						+	+	+			
C15	+						+	+	+			
C16	+						+	+	+			
C18	+						+	+	+			
C20	+						+	+	+			
C8	+						+	+	+			+
C4	+						+	+	+			+
C12	+						+	+	+			+
C24	+						+	+	+			+
C25	+						+	+	+			+
C3	+					+		+	+			
C10	+					+		+		+		
C11	+			+		+		+	+			
C2	+			+		+		+	+			
C26	+			+		+		+	+			
C6	+							+				
C19		+	+		+						+	
C7		+	+		+						+	
C22		+	+		+						+	
C17										+	+	
C1	+										+	+
Freq ^3^	0.84	0.12	0.12	0.12	0.12	0.20	0.56	0.80	0.72	0.08	0.24	0.24

^1^ The virus clones analysed were sorted according to the results obtained in a clustering analysis carried out with their genomic sequences (see Materials and Methods (Section 4) and Figure S2). ^2,3^ Same legend as in Table 1.

**Table 3:** Mutations present in the genomic sequences of the virus clones isolated from lineage L2 at transfer 15.

Virus Clone ^1^	Mutations Present at Least in Two Virus Clones ^2^												
	1312 G/A	1371 G/A	2223 G/A	2228 C/U	2236 G/A	2452 C/U	2776 U/C	3326 C/U	3359 U/C	3393 G/A	3402 U/C	3784 U/C	3879 C/A
C8	+			+			+	+					+
C3	+			+			+	+					+
C15	+			+			+		+				+
C9	+			+			+		+				+
C2				+			+						+
C1				+									
C10						+	+						
C4					+	+	+					+	
C14		+					+					+	
C5		+			+		+					+	
C7	+									+	+	+	
C6	+									+	+	+	
C11	+						+	+		+	+	+	
C13	+	+	+				+					+	
C12	+		+				+					+	
Freq ^3^	0.60	0.20	0.13	0.40	0.13	0.13	0.80	0.20	0.13	0.20	0.20	0.60	0.30

^1^ The virus clones analysed were sorted according to the results obtained in a clustering analysis carried out with their genomic sequences (see Materials and Methods (Section 4) and Figure S3). ^2,3^ Same legend as in Table 1.

**Table 4:** Mutations present in the genomic sequences of the virus clones isolated from lineage L2 at transfer 20.

Virus Clone ^1.^	Mutations Present at Least in Two Virus Clones ^2^								
	1088 A/G	1312 G/A	2228 C/U	2261 A/G	2776 U/C	3393 G/A	3402 U/C	3784 U/C	3879 C/A
C3	+	+	+		+				+
C1	+	+	+		+				+
C4	+	+	+		+				+
C5	+	+	+		+				+
C6	+	+	+		+				+
C8	+	+	+		+				+
C11	+	+	+		+				+
C9	+		+		+				+
C7	+		+		+				+
C10	+		+		+				+
C13	+		+		+				+
C14	+		+		+				+
C15	+		+		+				+
C2		+		+		+	+	+	
C12		+		+		+	+	+	
C16	+	+							
Freq ^3^	0.90	0.63	0.81	0.13	0.81	0.13	0.13	0.13	0.81

^1^ The virus clones analysed were sorted according to the results obtained in a clustering analysis carried out with their genomic sequences (see Materials and Methods (Section 4) and Figure S4). ^2,3^ Same legend as in Table 1.

## Data Availability

The datasets generated and/or analysed during the current study are available from the corresponding author on reasonable request.

## Supplementary Materials

The following are available online at https://www.mdpi.com/article/10.3390/ijms22136815/s1.
