# Supplementary material for: Intra-Population Competition during Adaptation to Increased Temperature in an RNA Bacteriophage
Source: Int J Mol Sci. 2021 Jun 24;22(13):6815. doi: 10.3390/ijms22136815 (PMC8268601; doi:10.3390/ijms22136815)
Supplement: Supplementary file 1 [file Figure S4.pdf › ijms-1235742-supplementary/Figure S4.pdf]

Figure S4. Clustering analysis of the genomic sequences corresponding to virus clones isolated from L2 at transfer number 20

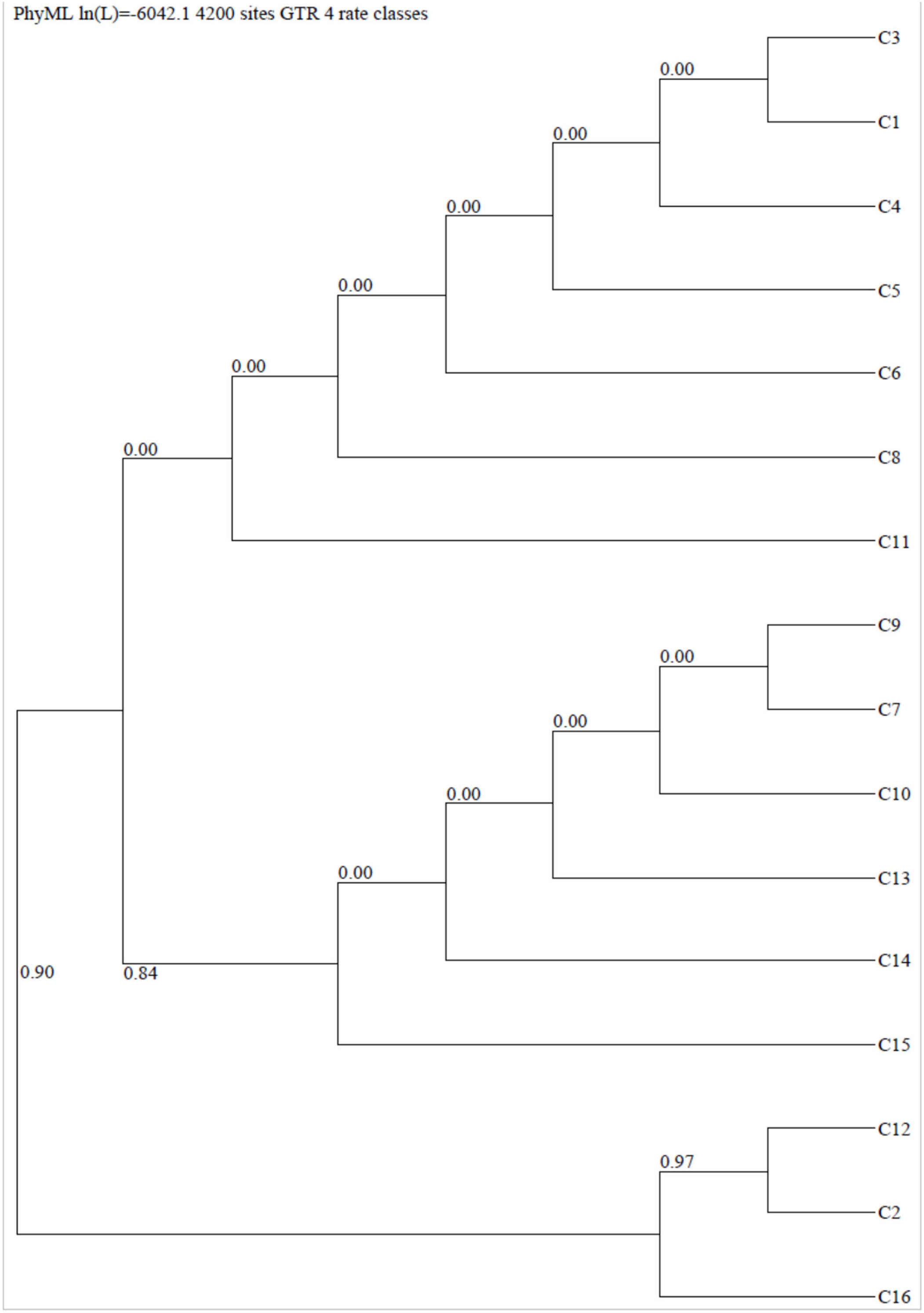

Mutations present in the sequence of each virus clone are shown in Table 4 (mutations repeated in more than one genome) and in Supplementary Table S2 (mutations present in single virus clones).
