# Supplementary material for: Intra-Population Competition during Adaptation to Increased Temperature in an RNA Bacteriophage
Source: Int J Mol Sci. 2021 Jun 24;22(13):6815. doi: 10.3390/ijms22136815 (PMC8268601; doi:10.3390/ijms22136815)
Supplement: Supplementary file 1 [file ijms-22-06815-s001.zip › ijms-1235742-supplementary/Table S1.pdf]

Table S1. Virus titers (pfu/mL) obtained in a control experiment carried out to check the possibility of cross-contamination between lineages

Transfer number	Experimental sample 1[1]	Experimental sample 2[1]	Negative Control 1[2]	Negative control 2[2]
1	2.3×10^{9}	3.0×10^{9}	0	0
2	2.5×10^{9}	1.5×10^{9}	0	0
3	3.2×10^{9}	1.2×10^{9}	0	0
4	9.6×10^{9}	3.7×10^{9}	0	0
5	2.5×10^{10}	4.2×10^{10}	0	0
6	3.0×10^{10}	1.9×10^{9}	0	0
7	2.4×10^{10}	3.3×10^{9}	0	0
8	1.1×10^{10}	9.0×10^{9}	0	0
9	6.6×10^{9}	1.6×10^{10}	0	0
10	5.5×10^{9}	8.5×10^{9}	0	0
11	6.0×10^{9}	2.5×10^{10}	0	0
12	1.2×10^{10}	1.4×10^{10}	0	0
13	8.9×10^{9}	9.2×10^{9}	0	0
14	2.3×10^{10}	7.3×10^{9}	0	0
15	3.5×10^{10}	4.1×10^{10}	0	0

[1]The experimental samples were initiated by infecting 500 μL of bacteria in exponential phase with 10^{7} pfu (in 100 μL of Phage Buffer) of a clonal virus obtained upon expression of pBRT7Qβ in a final volume of 1 mL completed with NB. Samples were incubated at 43 °C, and processed as described in Materials and Methods. Each new transfer was carried out with 100 μL of an 1:10 dilution of the previous supernatant. Proper dilutions were also plated to determine the virus yields.

[2]Negative controls were performed in the same way as the experimental samples but in the absence of virus. In this case, each new transfer was initiated with 100 μL of the previous undiluted supernatant. Other 100 μL were plated to check for the formation of lytic plaques.
