# Supplementary material for: Intra-Population Competition during Adaptation to Increased Temperature in an RNA Bacteriophage
Source: Int J Mol Sci. 2021 Jun 24;22(13):6815. doi: 10.3390/ijms22136815 (PMC8268601; doi:10.3390/ijms22136815)
Supplement: Supplementary file 1 [file ijms-22-06815-s001.zip › ijms-1235742-supplementary/Table S2.pdf]

Table S2. Additional mutations present in the virus clones shown in Tables 1-4

Population	Virus clone	Mutations[1]
L1 Transfer 10	C12	A3153C U3520C
	C13	A2092G
	C3	U1652C U2776C
	C9	U3149C U3581C
	C6	U1026C C3659U
	C2	A1149G
	C16	U1463C U2847C
	C7	G2223A
	C11	C2228U A3638G
	C5	G2906A
	C4	G3041A
L1 Transfer 15	C9	G1820A
	C5	G2636A
	C8	G2791A
	C4	C3893U
	C2	U2006C
	C6	A2222C A4070G
	C19	A1079G
	C17	A2261G
	C1	U1025C C2201U
L2 Transfer 15	C1	U2776G U3122C
	C10	A1088G G1773U
	C4	U1155C
	C7	U2847C A3997G
	C6	U3902C
L2 Transfer 20	C3	U1451C
	C6	U722C U768C
	C11	G4101A
	C9	C2138U
	C13	G1691A C2210U U3842C
	C14	C2519U U2954C
	C15	A3824G
	C12	G1371A
	C16	C1625U C1649U G1820A U2559C C3201A C3903A

[1]Mutations marked in red have been identified in previous studies in which Qβ adapted to high temperature [40-44].
