# Supplementary material for: Intra-Population Competition during Adaptation to Increased Temperature in an RNA Bacteriophage
Source: Int J Mol Sci. 2021 Jun 24;22(13):6815. doi: 10.3390/ijms22136815 (PMC8268601; doi:10.3390/ijms22136815)
Supplement: Supplementary file 1 [file ijms-22-06815-s001.zip › ijms-1235742-supplementary/Figure S1.pdf]

Figure S1. Clustering analysis of the genomic sequences corresponding to virus clones isolated from L1 at transfer number 10

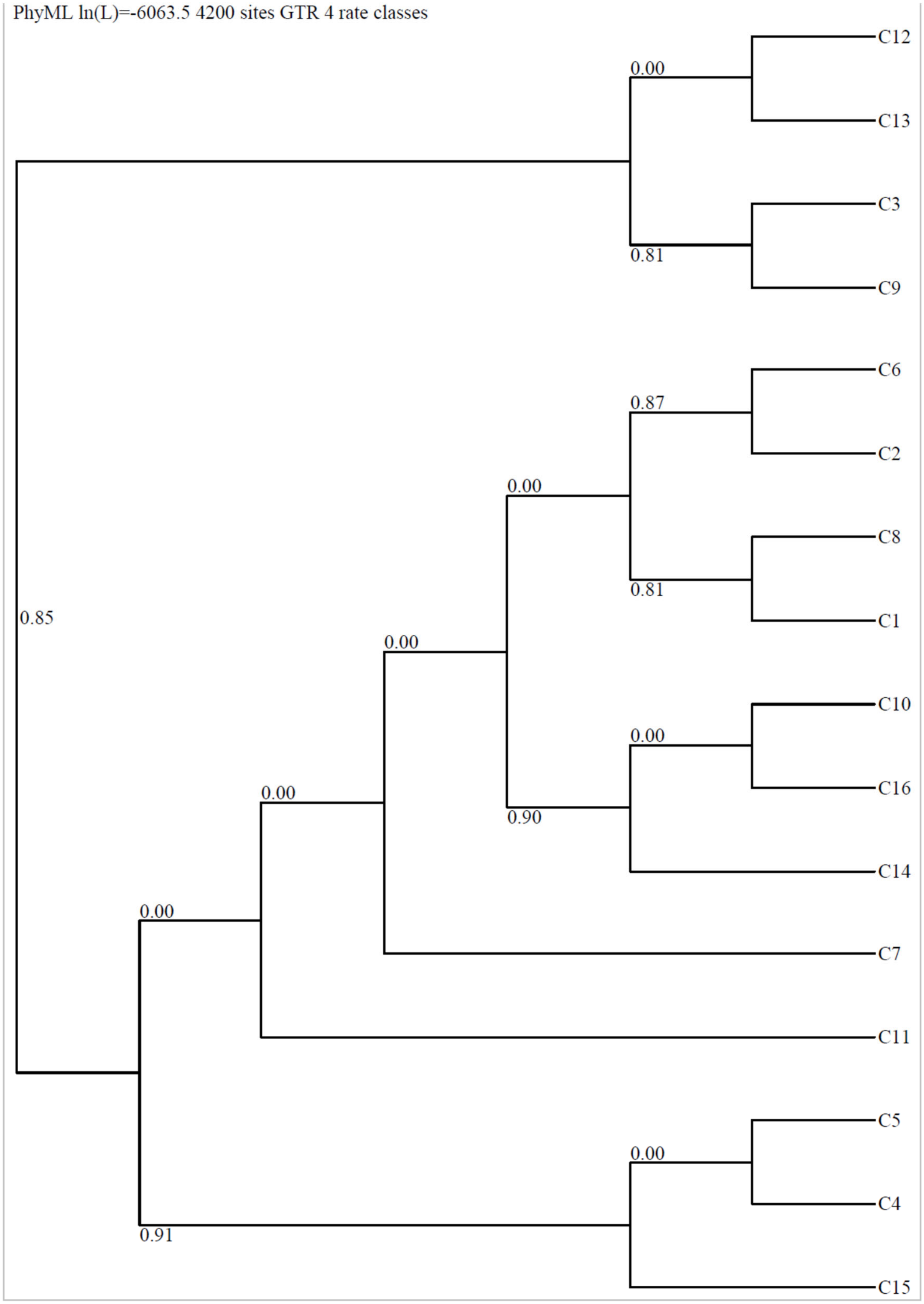

Mutations present in the sequence of each virus clone are shown in Table 1 (mutations repeated in more than one genome) and in Table S2 (mutations present in single virus clones).
